# ProteinNetworkSight: a user-friendly platform for transforming co-expression patterns into actionable therapeutic insights through interactive network visualization

**DOI:** 10.1093/nar/gkag477

**Published:** 2026-05-14

**Authors:** Omri Nahor, Nitzan Migdal, Ayelet Gibli, Tohar Tsvitman, Aviv Eldad, Shell Raveh, Gil Polinovski, Deema Zaid, Nataly Kravchenko-Balasha, Noa E Cohen

**Affiliations:** Software Engineering Department, School of Software and Electrical Engineering, Azrieli College of Engineering, Jerusalem 9103501, Israel; Software Engineering Department, School of Software and Electrical Engineering, Azrieli College of Engineering, Jerusalem 9103501, Israel; Software Engineering Department, School of Software and Electrical Engineering, Azrieli College of Engineering, Jerusalem 9103501, Israel; Software Engineering Department, School of Software and Electrical Engineering, Azrieli College of Engineering, Jerusalem 9103501, Israel; Software Engineering Department, School of Software and Electrical Engineering, Azrieli College of Engineering, Jerusalem 9103501, Israel; The Institute of Biomedical and Oral Research, The Hebrew University of Jerusalem, Jerusalem 9103401, Israel; The Institute of Biomedical and Oral Research, The Hebrew University of Jerusalem, Jerusalem 9103401, Israel; The Institute of Biomedical and Oral Research, The Hebrew University of Jerusalem, Jerusalem 9103401, Israel; The Institute of Biomedical and Oral Research, The Hebrew University of Jerusalem, Jerusalem 9103401, Israel; Software Engineering Department, School of Software and Electrical Engineering, Azrieli College of Engineering, Jerusalem 9103501, Israel

## Abstract

ProteinNetworkSight (https://proteinnetworksight.jce.ac) addresses a pervasive bottleneck in modern systems biology: the inability to simultaneously analyze multiple feature vectors generated by quantitative techniques—such as machine learning, deep learning, or statistical modeling—that provide series of patterns in a dataset. Modern computational pipelines, ranging from PCA to deep autoencoders, rarely identify a single gene list; instead, they extract a series of distinct patterns representing diverse patient subgroups or independent components. Current web servers are ill-equipped for this high-dimensional reality, forcing researchers to analyze vectors one-by-one or merge them into a static consensus, obliterating unique topological signatures. ProteinNetworkSight introduces a novel web server architecture for simultaneous multi-pattern analysis. Unlike standard tools, our server accepts multi-column tables and transforms every input vector into a discrete, interactive protein–protein interaction network in a single run. This batch vector architecture allows side-by-side visualization of distinct topologies, preserving disease heterogeneity. Furthermore, the server enables prescriptive intervention by calculating a composite perturbation score to identify key protein nodes specific to each pattern. By mapping FDA-approved anti-cancer drugs to these targets, it facilitates the rapid design of personalized combinatorial therapies.

## Graphical Abstract

**Graphical Abstract:**
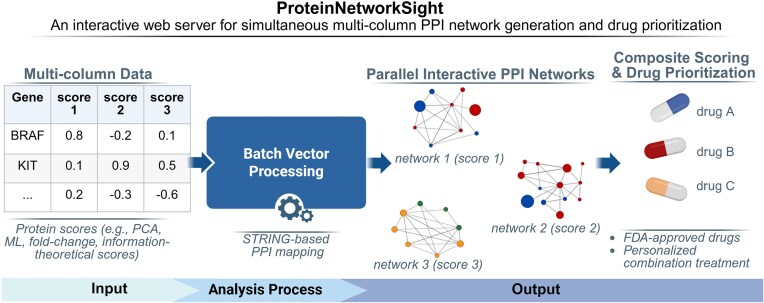

## Introduction

Precision medicine represents a paradigm shift in treating complex diseases such as cancer and heart disease by tailoring therapies to specific patient subgroups [1–5], moving beyond the limitations of a one-size-fits-all approach. The field’s progress is fueled by large-scale omics data and sophisticated computational techniques. Recognizing that single biomarkers often fail to capture the intricate molecular landscape of these diseases, the research community has increasingly adopted network analysis [6], to investigate the complex interplay of genomic and proteomic alterations [7–10].

Numerous computational tools have been developed to analyze patient-specific molecular networks, including advanced machine/deep learning [11–14], network-based methods [15, 16], and information-theoretic approaches [17–21]. These methodologies typically generate multiple numeric vectors or patterns (e.g. principal components 1, 2, etc.), each representing the contribution of individual genes or proteins to distinct biological states, time points, or independent components. Consequently, the analytical output is inherently multi-dimensional rather than a single unified gene list.

Current web servers lack the architecture to analyze these “batches” simultaneously. Instead, they require researchers to analyze these vectors one-by-one, a disjointed and labor intensive process that prevents the immediate comparison of independent biological signals. To address this gap, we introduce ProteinNetworkSight (freely accessible at https://proteinnetworksight.jce.ac), a novel user-friendly web platform. While existing resources like STRING provide essential protein–protein interaction data [22], ProteinNetworkSight introduces a novel batch-vector-to-parallel-network architecture that treats multi-column quantitative outputs as a unified topological landscape, generating multiple interactive protein-protein interaction (PPI) networks simultaneously.

Importantly, the platform integrates an approved anti-cancer drug database [23], providing immediate insights into how identified networks may be therapeutically modulated. Notably, the “final score” serves as a multi-pattern prioritization metric. While standard tools typically analyze gene/protein lists in isolation, this metric prioritizes proteins based on their topological and interaction-based significance within patient-specific signaling landscapes.

The final score is computed for each individual node, integrating both the number of interacting partners within each specific network vector and their respective interaction weights (derived from STRING confidence scores). This approach identifies proteins that might not exhibit the highest weight within a single isolated pattern, yet achieve a high cumulative score due to their robust local connectivity. Furthermore, because a single tissue or system is often characterized by several co-active patterns, the server compares these key driver nodes across the entire signaling landscape. By examining the combination of these most optimal nodes across multiple patterns, the platform moves beyond static visualization toward prescriptive system pharmacology. This allows for a superior examination of drug combinations, linking FDA-approved drugs directly to the underlying network topologies to facilitate highly targeted therapeutic interventions.

## Methods

### Software workflow and user guidance

**Figure 1.**
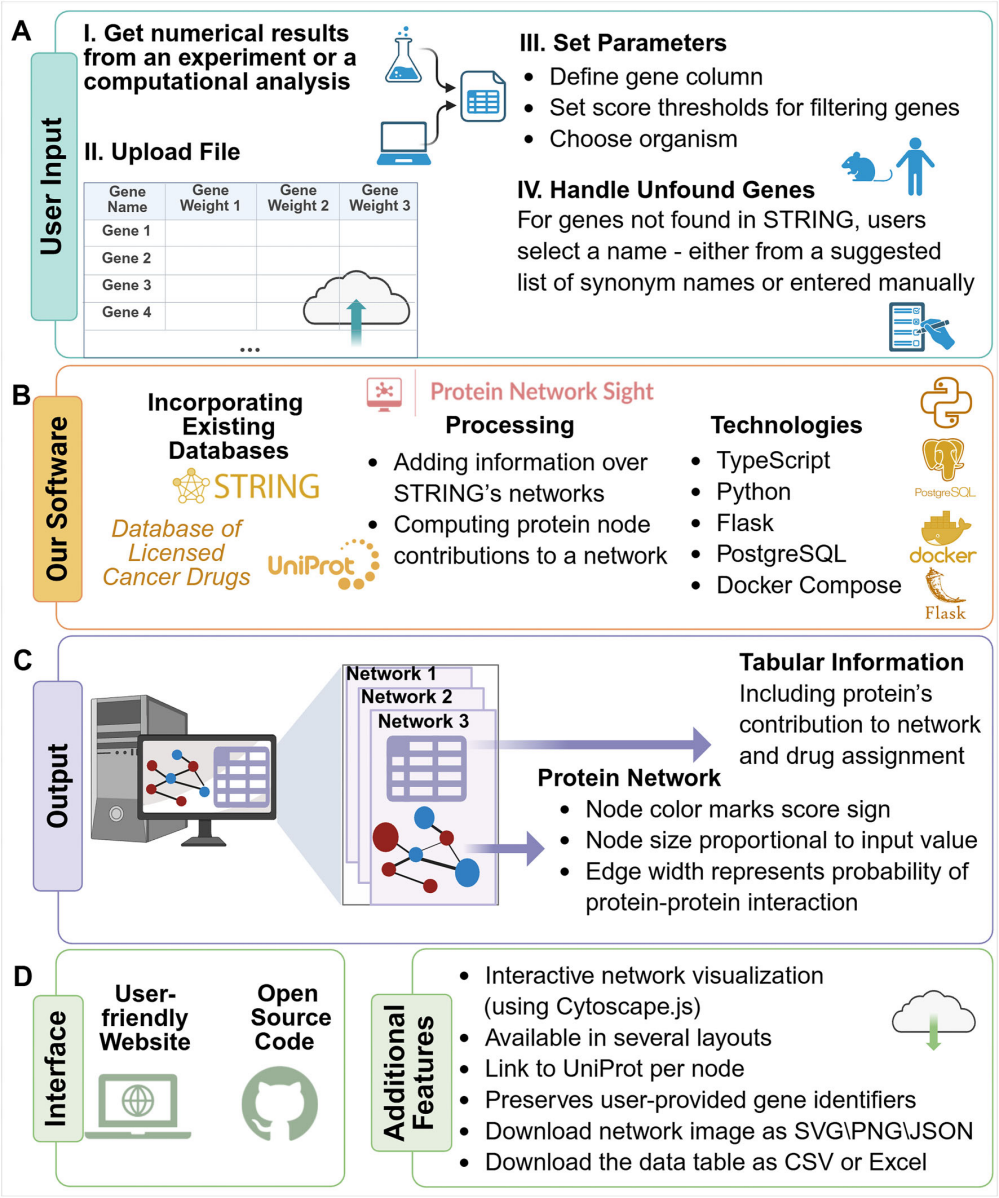
Overview of the tool. (**A**) User input is comprised of several stages: (I) uploading data file, (II) configuring parameters (e.g. interaction score threshold as defined by STRING, protein/genes score threshold per column (to define which genes will be included in a network/s), and choosing organism), (III) for each gene name not found in STRING, user is presented with synonym name(s) recognized by STRING or asked to manually enter an alternative name. Users upload data files that contain scores per gene/protein (rows represent genes/proteins, columns represent scores). Scores can be either values showing a gene/protein contribution to a certain pattern as acquired by machine learning/statistical or any other sort of computational analysis, or a simple fold change in a specific experimental condition. (**B**) The tool processes the data while integrating information from existing databases (STRING, cancer drugs database, UniProt). (**C**) The output comprises of a protein network per column of gene/protein scores, as well as a tabular representation with additional information (including node degree, weighted node degree, the final score, and associated drugs). (**D**) The tool is available both as a web-based application and as an open-source. Created in BioRender. Cohen, N. (2026) https://BioRender.com/d1q4zbk.

**Figure 2.**
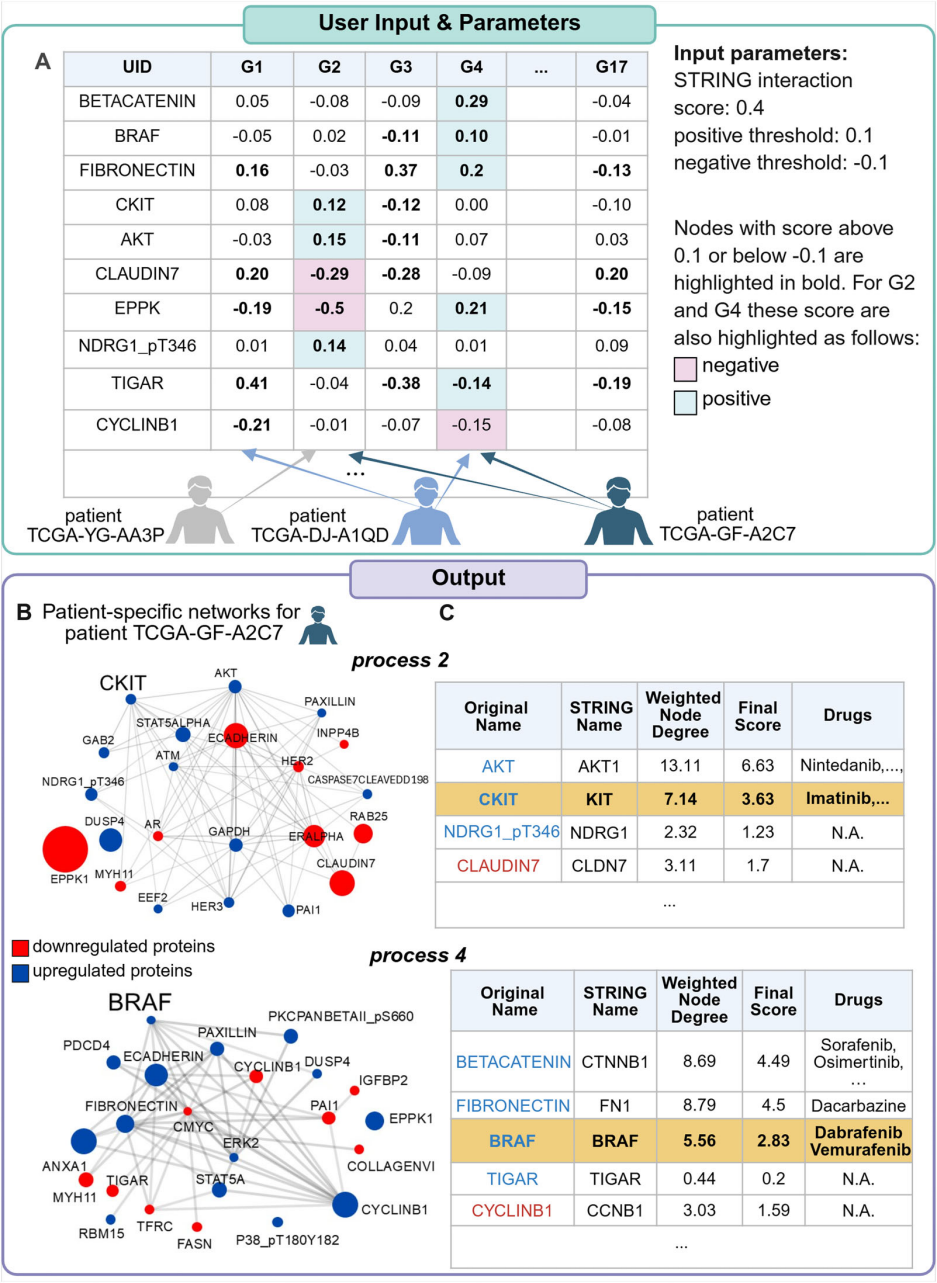
Tool demonstration using real-world proteomics data. (**A**) The example input file, extracted from supplementary data 1 (tab G [27]), illustrates the required data format. The column headers define the gene/protein identifiers (UID) and the numerical score prefixes (G). The tool filters proteins based on user-defined thresholds; in this case, a STRING interaction score of 0.4 was applied. To focus on proteins with the highest network impact [27], thresholds were set at >0.1 for positive scores and <−0.1 for negative scores, with the organism designated as *Homo sapiens*. For visualization purposes, only the 9 from the 216-protein dataset are displayed in the input panel for 5 score columns out of 17 (G). Nodes with scores above 0.1 (referred to as “positive scores”) or below −0.1 (negative score) are highlighted in bold. Negative and positive scores are highlighted in light red and light blue, respectively, for G2 and G4. Since every individual patient in these large-scale cohorts is defined by a unique combination of co-expression patterns, ProteinNetworkSight can generate similar diagnostic and therapeutic visualizations for any patient within the dataset; panels (B) and (C) illustrate one such representative example. Results for patient TCGA-GF-A2C7 (e.g. columns G2 and G4) are presented as interactive networks (**B**) and data tables (**C**). These outputs detail calculated metrics-including node degree (not shown in the figure), weighted node degree, and final scores alongside information regarding linked direct/indirect pharmacological inhibitors; specifically, the output tables for process 2 and process 4 display 3 out of 13 upregulated proteins co-expressed with c-Kit and 4 out of 14 upregulated proteins co-expressed with BRAF, respectively. In step 4, instead of the original name that was not identified in STRING, the matching STRING names were entered as follows: KIT for CKIT, CASP7 for CASPASE7CLEAVEDD198, CLDN7 for CLAUDIN7, CDH1 for ECADHERIN, ESR1 for ERALPHA, STAT5A for STAT5ALPHA, CTNNB1 for BETACATENIN, MYC for CMYC, COL6A1 for COLLAGEN VI, CCNB1 for CYCLINB1, CDH1 for ECADHERIN, PRKCA for PKCALPHA_pS657, and PRKCB for PKCPANBETAII_pS660. Created in BioRender. Cohen, N. (2026) https://BioRender.com/ceytlpq.

This website is free and open to all users and there is no login requirement. Detailed operating instructions are provided through an on-site tutorial. In addition, a contextual panel on the left side of each page provides explanations of the current step and the available features. The server accepts user data in Excel, CSV, or TSV formats. As illustrated in Figs 1 and 2, uploaded files are processed by applying user-defined thresholds (see the tutorial for more details) to filter genes and convert numerical vectors into interactive protein–protein interaction networks. For each column, users define score thresholds that determine which proteins are included in the network. Proteins whose scores fall within the defined range are excluded, thereby focusing the analysis on proteins with the strongest positive or negative contributions. Each column is processed independently, enabling parallel network generation within a single automated workflow. Nodes are colored according to the sign of the input scores (by default, nodes are colored blue for positive values and red for negative values, but the color scheme can be configured by the user), while edge widths correspond to the STRING interaction confidence score. Depending on the user-defined analysis context, colors may represent correlations/anti-correlations or increases/decreases. Each protein node also includes a direct link to its corresponding UniProt record, allowing users to easily access additional protein annotations and functional information.

The output table shows for each gene or protein its connectivity degree (node degree), the “weighted node degree” (computed as the sum of the node’s link weights), and the final score, defined as the average between the node size (the absolute value of the input score) and its weighted node degree. This metric integrates the biological contribution of each protein with its topological importance in the network, enabling prioritization of proteins with both high scores and extensive connectivity. The website also provides a downloadable example file (whose output is visualized in Supplementary Fig. S1), allowing users to verify formatting requirements and explore the platform’s features.

### Incorporating existing databases (Fig. 1B)

Node-to-node links as well as links’ scores are derived from a local copy of STRING’s DB (https://string-db.org/, version 12.0). The database is managed using pgAdmin (https://www.pgadmin.org/) over PostgreSQL (version 16.11, PostgreSQL Global Development Group), with indexing applied to the larger tables to enable efficient query performance. Information on protein–drug associations was obtained from the Database Cancer Drugs of Anticancer Fund [23]. The publicly available text file (downloaded from https://data.tp53.org.uk/cancerdrugs.php; last database update: Dec 16, 2025) was downloaded and parsed into the required format using the Pandas library (Pandas 3.0.1) in Python. Links to UniProt entries [24] are also provided to allow users to access additional protein annotations.

### Server-side implementation (Fig. 1B)

All computational analyses and data processing steps (including input parsing and score calculations) were implemented in Python (version 3.11.15, Python Software Foundation). The backend web application was developed using the Flask microframework (version 3.1.3). To improve performance and ensure separation between development and production environments, the backend is deployed within a Docker container (version 28.0.1) and NGINX reverse proxy is used to support load balancing, caching, and security. Cloudflare DNS is used for domain management and security protection.

### Website development

The frontend application of ProteinNetworkSight (https://proteinnetworksight.jce.ac/) was developed using React and TypeScript. Protein–protein interaction networks are rendered using Cytoscape.js (version 3.31.0), which enables dynamic graph manipulation and interactive visualization. To facilitate exploration of the generated networks, the platform provides several layout options implemented through Cytoscape.js. In addition, we introduce a custom layout algorithm termed LCSL, which positions the node with the highest cumulative interaction weight at the center of a cluster, while its direct neighbors are arranged in a spiral pattern according to the same metric. SVG export functionality was implemented to allow offline use of calculated network data. In addition to PNG images, networks can also be downloaded in JSON format for further editing in Cytoscape (https://cytoscape.org/) or as SVG files for vector-based image editing (Fig. 1D).

## Results

ProteinNetworkSight converts multi-column numeric inputs into interactive PPI network maps (Fig. 1A–C). Unlike conventional tools that process gene lists as isolated queries, our server accepts a multi-column table and efficiently transforms every numeric vector into a discrete, interactive network within a single run (Fig. 1A). This batch vector architecture is specifically designed for high-dimensional inputs in which numeric values represent quantitative metrics (the outputs of upstream computational analyses) such as principal component loadings, information-theoretic weights, or fold changes. Rather than collapsing distinct signals into a consensus map, ProteinNetworkSight generates a dedicated network for each column simultaneously (Fig. 1C). This parallel construction preserves the unique topological signatures of each experimental condition and maintains the molecular heterogeneity inherent in complex biological datasets.

Protein or gene scores derived from upstream analyses are intuitively represented by node size (Fig. 1C), while protein–protein correlations and anti-correlations are indicated through distinct color coding. Interaction data from the STRING database (Fig. 1B) are automatically incorporated to connect co-expressed proteins based on known and predicted PPIs. A central feature of the platform is the calculation of a “final score” (labeled as such on the website, Methods) for each node across the simultaneously generated networks. This metric mathematically integrates the connectivity degree—the number of partners a protein has—with its biological relevance, such as user-provided weights or significance scores. By identifying key protein nodes characterized by both high weights and a high number of partners, the tool enables an informed prioritization of potential therapeutic targets within each specific pattern. Several network layout options, including circle, concentric, grid, and a custom layout algorithm (LCSL), further assist users in visually identifying highly connected and biologically significant proteins (Fig. 1C and D).

Importantly, proteins with the highest input scores are not necessarily the most central nodes in the network. A protein may receive a high contribution score (weight of a protein within a specific biological pattern) yet participate in only a limited number of interactions, resulting in a relatively low final score. Conversely, proteins with moderate input scores but extensive connectivity may achieve higher final scores, highlighting their potential importance as key regulatory hubs and therapeutic targets within the network.

An additional distinctive feature of the platform is the direct integration of a curated database of licensed anticancer drugs (Fig. 1B). Unlike generic enrichment tools utilizing text mining, ProteinNetworkSight maps inhibitors directly onto prioritized topological vulnerabilities, enabling real-time visualization of drug–target-network interactions. In all cases, the platform suggests both direct and indirect pharmacological inhibitors based on documented literature. For each suggested agent, the server provides comprehensive metadata, including drug indications, DrugBank ID, ChEMBL ID, and ATC (Anatomical Therapeutic Chemical) Code, allowing for the systematic evaluation of intervention strategies tailored to patient-specific networks. This provides a comprehensive computational suggestion for disrupting specific disease modules and supports personalized decisions.

### Implementation

The pipeline begins with the upload of a multi-column file (step 1) in which rows represent genes or proteins and columns represent their quantitative scores. In step 2, users may define analysis parameters, including STRING’s interaction confidence threshold and column-specific score thresholds that determine which proteins are included in the networks (Fig. 1A and tutorial).

Gene nomenclature inconsistencies can lead to data loss in automated pipelines due to synonym variability. To address this issue, the platform incorporates automated synonym matching and fuzzy logic to recover unrecognized identifiers. Because interaction data are derived from STRING, gene names must be mapped to the identifiers used in the STRING database. When a protein name is not directly recognized, the platform suggests potential STRING-recognized synonyms to assist the user (step 3).

In step 4, users may optionally resolve unmatched identifiers; otherwise, proteins without a recognized match are excluded from the analysis. Each identifier entered by the user is immediately validated against the STRING database, and an informative message is displayed to indicate whether a valid match has been found (Fig. 1A).

The output page includes a “Missing Nodes” button listing proteins for which neither an automatic synonym nor a user-provided match was identified, and a “Nodes Worth Reviewing” button listing proteins whose identifiers were assigned during step 3. This workflow minimizes identifier loss and maximizes interactome coverage.

The platform also supports protein names that include modification annotations and automatically extracts the base gene name (e.g. removing phosphorylation suffixes such as *pT346* from *NDRG1_pT346*) before attempting identifier matching. Once a match is identified (e.g. *NDRG1* for *NDRG1_pT346* or *CLDN7* for *CLAUDIN7*), the original annotation is restored in the final visualization and output tables. This approach preserves biologically relevant modification information while ensuring compatibility with STRING identifiers. If needed, the network visualization can also display the canonical STRING identifiers.

In addition, the platform provides a downloadable file containing the original input together with the resolved STRING identifiers. Users may edit this file to remove unmatched proteins or replace custom gene names with standardized STRING identifiers, and then re-upload the updated file to continue the analysis without repeating the identifier-matching step. Networks corresponding to all input vectors can also be downloaded in bulk as a compressed ZIP archive.

**Functional illustration: pan-cancer multi-pattern resolution and inter-tumor heterogeneity** A major advantage of ProteinNetworkSight is its batch vector architecture, which allows multiple pattern vectors to be uploaded and processed simultaneously in a single computational run. This architecture is particularly critical for clinical translation; because recent studies have shown that an individual patient’s molecular profile is often defined by a combination of a few pathways/co-expression patterns [5, 25–27], the ability to resolve these patterns concurrently is essential. By evaluating these patient-specific combinations side-by-side (Fig. 2A), researchers can directly resolve inter-tumor heterogeneity and pinpoint the exact therapeutic vulnerabilities active in a patient at that moment. This allows for the immediate design of personalized combinatorial therapies tailored to the unique set of active drivers, even for patients sharing the same clinical diagnosis.

To demonstrate the platform’s utility in interpreting clinical data, we analyzed a dataset from a study that identified 17 distinct protein–protein co-expression patterns (named ongoing biological processes) across 353 skin cutaneous melanoma samples [27]. Each pattern corresponds to a separate quantitative vector and is therefore translated by ProteinNetworkSight into an independent interactive network within a single computational run (Fig. 2A). In this framework, all 17 networks are generated simultaneously, together with associated drug annotations, enabling rapid comparative analysis across patterns. Each patient-specific tumor profile comprised a unique combination of 2–3 processes out of the 17 identified ([27], Fig. 2A). This parallel processing approach facilitates pattern-specific target prioritization without requiring sequential network reconstruction.

As a representative example, we focus on BRAF-mutated melanoma patient TCGA-GF-A2C7 [27]. This specific patient is characterized by the simultaneous activities of process 2 and process 4 (Fig. 2B). This finding is significant because it moves beyond a single-driver model. It suggests that the tumor’s survival is dependent on a patient-specific set of ongoing molecular networks. By isolating these 2 processes out of the 17 identified, the platform allows for a targeted intervention that addresses the actual active drivers in this individual’s molecular signature, rather than a generic treatment approach.

For this BRAF-mutated melanoma patient, conventional clinical practice would typically prioritize anti-BRAF therapy. The analysis by Vasudevan *et al*. suggests that both biological processes, process 4 and process 2, should be targeted concurrently [27], as both networks coexist within the tumor. Data visualization and analysis using ProteinNetworkSight reveal that while BRAF in process 4 exhibits a relatively low node weight, it maintains a high final score due to its high connectivity; it ranks 5th out of 14 blue-colored proteins that are co-expressed and upregulated alongside BRAF (Fig. 2C).

Notably, other proteins in these networks present higher final scores, such as AKT (AKT1 in STRING) in process 2 (final score 6.63) or bCatenin (CTNNB1 in STRING) and Fibronectin (FN1 in STRING) in process 4 (final scores >4.49). Nevertheless, a clinician may prioritize BRAF (process 4) and c-KIT (ranked 2nd out of 14 blue-colored proteins that are co-expressed and upregulated alongside c-KIT in process 2) for this specific case. This prioritization is due to the fact that these two proteins possess FDA-approved, highly specific targeted therapies for melanoma (e.g. vemurafenib/dabrafenib for BRAF and imatinib for c-KIT). Utilizing these approved drugs in combination offers a clear path for clinical implementation that is significantly more feasible than targeting undruggable hubs or using non-specific inhibitors, even though this exact combination is not yet standard clinical practice.

Additional prioritized targets and their corresponding approved drugs are presented in the Results panel (Fig. 2C). In any case, a clinician or researcher can select alternative drugs for other targets within each process, provided a drug exists that targets the protein directly or inhibits it indirectly (e.g. via upstream targets), as shown in the table (Fig. 2C). This flexibility allows the user to choose which drugs from different processes (e.g. process 2 and 4) to combine for a tailored intervention based on drug availability, cost, or potential drug–drug interactions. By presenting the node degrees and the final score values alongside actionable drug data, the platform supports the systematic identification of candidate nodes within patient-specific molecular signatures and facilitates the exploration of concurrent network inhibition.

To further illustrate the platform’s capacity to exploit large-scale datasets and broader FDA drug applications, we utilized a pan-cancer dataset from Flashner-Abramson *et al*., which analyzed 3 467 tumors across 11 different cancer types [28]. This study resolved the molecular heterogeneity of these tumors into 17 distinct protein network patterns, with individual patient typically harboring a unique combination of 1–4 active subnetworks.

For example, evaluating two colon adenocarcinoma (COAD) patients reveals completely divergent multi-pattern signaling networks. Patient TCGA-CM-6171 harbors a combination of active processes 3, 7, and 10 (Supplementary Fig. S3D). Instead of prioritizing a single generic biomarker, ProteinNetworkSight concurrently generates interactive networks for these specific processes and calculates a composite final score for their nodes to identify central vulnerabilities. For process 3, while no direct drug was initially identified, the platform’s prioritization of GAPDH (the node with the highest final score) allows for the selection of tamoxifen, which research suggests can indirectly inhibit glycolysis [29]. In process 10, AKT was identified with the highest final score and can be targeted by the recently approved inhibitor Capivasertib [30]. For process 7, although EGFR (Epidermal Growth Factor Receptor) is ranked third by final score, it remains a high-priority target due to the availability of direct approved inhibitors such as osimertinib (Supplementary Fig. S3E).

Conversely, a second COAD patient, TCGA-A6-5664, exhibits a distinct network topology driven solely by process 2. In this case, ProteinNetworkSight identifies VEGFR (Vascular Endothelial Growth Factor Receptor) as a key node; despite ranking third in terms of final score, it is prioritized because it has a direct approved inhibitor, Ramucirumab (Supplementary Fig. S3B and C). This demonstrates that simultaneous evaluation of parallel PPI networks, coupled with flexible drug mapping, enables researchers - and potentially clinicians in the future - to prioritize highly personalized FDA therapies that traditional baseline methods might overlook.

All parameters used for network generation, including score thresholds and interaction confidence values, are recorded in the output tables and downloadable files as shown in Fig. 2 and Supplementary Figs S1 and S3. This ensures that analyses performed with ProteinNetworkSight can be reproduced and independently verified.

## Discussion

The interpretation of output from advanced computational methods in biology, often represented as numeric vectors of gene or protein scores, is essential for translating research findings into tangible experimental or therapeutic strategies. Current system biology methods frequently generate multiple complex molecular patterns; however, tools capable of analyzing these high-dimensional outputs simultaneously remain limited, resulting in a persistent analytical bottleneck. Several established resources support the visualization and exploration of protein interaction networks. STRING provides a comprehensive resource of known and predicted protein–protein interactions and enables users to generate interaction networks from gene lists. Cytoscape is a widely used desktop platform for constructing and visualizing biological networks through customizable workflows and plugins. However, these tools generally require sequential analysis of individual gene lists or manual workflow construction. In addition, STRING-based visualizations typically display canonical identifiers recognized by the database (e.g. the alias LKB1 may appear as STK11), which may differ from the original labels used in the user’s dataset.

Unlike traditional workflows that force a “Single Gene-List” paradigm, ProteinNetworkSight introduces a batch-vector-to-parallel-network methodology. This architectural shift is essential for current systems biology, where biological truth often resides in the combination of multiple co-active patterns rather than a single isolated list. By treating multi-column quantitative tables as a unified topological landscape, the platform preserves the unique signatures of each pattern—whether derived from complex machine learning scores or simple expression changes—while maintaining consistency with the user’s original dataset annotations.

Beyond mere visualization, the tool intuitively displays protein significance, co-expression relationships, and protein–protein interactions within an integrated environment. The final score metric enables researchers to move past raw weights by identifying “hidden hubs”—nodes characterized by both high biological significance and high interaction density. When coupled with the integrated database of licensed anticancer drugs, this topological analysis transforms a labor-intensive manual process into a rapid, prescriptive workflow for evaluating intervention strategies.

As demonstrated in our examples, this parallel evaluation can suggest synergistic combinations, such as anti-KIT and anti-BRAF, that might be overlooked in standard clinical settings where biomarkers are analyzed in isolation. Openly available as both a web server and source code, ProteinNetworkSight provides the scientific community with a scalable framework to accelerate the development of personalized combinatorial therapies.

## Supplementary Material

gkag477_Supplemental_File

## Data Availability

Code relevant to this manuscript can be found at https://github.com/cohenoa/ProteinNetworkSight and https://zenodo.org/records/19881700 (DOI: 10.5281/zenodo.19881700).

## References

[1] Mai N, Fernandez N, Drilon A et al. Precision oncology: 2025 in review. Cancer Discov. 2025;15:2414–21. 10.1158/2159-8290.CD-25-1784. 41327967

[2] Gonzalez-Angulo AM, Hennessy BTJ, Mills GB. Future of personalized medicine in oncology: a systems biology approach. J Clin Oncol. 2010;28:2777–83. 10.1200/JCO.2009.27.0777. 20406928 PMC2881854

[3] Raufaste-Cazavieille V, Santiago R, Droit A. Multi-omics analysis: paving the path toward achieving precision medicine in cancer treatment and immuno-oncology. Front Mol Biosci. 2022;9:962743. 10.3389/fmolb.2022.962743. 36304921 PMC9595279

[4] Abdelaziz EH, Ismail R, Mabrouk MS et al. Multi-omics data integration and analysis pipeline for precision medicine: systematic review. Comput Biol Chem. 2024;113:108254. 10.1016/j.compbiolchem.2024.108254. 39447405

[5] Alkhatib H, Conage-Pough J, Roy Chowdhury S et al. Patient-specific signaling signatures predict optimal therapeutic combinations for triple negative breast cancer. Mol Cancer. 2024;23:1–7. 10.1186/s12943-023-01921-9. 38229082 PMC10790458

[6] Ozturk K, Dow M, Carlin DE et al. The emerging potential for network analysis to inform precision cancer medicine. J Mol Biol. 2018;430:2875–99. 10.1016/j.jmb.2018.06.016. 29908887 PMC6097914

[7] Yaffe MB, VanHook AM. *Science Signaling* Podcast for 14 March 2017: the Cancer Moonshot. Sci Signal. 2017;10:eaan1418. 10.1126/scisignal.aan1418. 28292953

[8] Lam FC, Yaffe MB. Kicking genomic profiling to the curb: how re-wiring the phosphoproteome can explain treatment resistance in glioma. Cancer Cell. 2016;29:435–6. 10.1016/j.ccell.2016.03.022. 27070697

[9] Petak I, Kamal M, Dirner A et al. A computational method for prioritizing targeted therapies in precision oncology: performance analysis in the SHIVA01 trial. npj Precis Oncol. 2021;5:59. 10.1038/s41698-021-00191-2. 34162980 PMC8222375

[10] Daher-Ghanem N, Sharon S, Tao D et al. The responses of HNSCC patients to immunotherapy are shown by two novel co-expression patterns. npj Precis Oncol. 2025;9:1–10. 10.1038/s41698-025-00983-w. 40596712 PMC12217146

[11] Cohen S . The basics of machine learning: strategies and techniques. In: Cohen S (ed.), Artificial Intelligence and Deep Learning in Pathology. Amsterdam, The Netherlands: Elsevier Health Sciences, 2021, 13–40. 10.1016/B978-0-323-67538-3.00002-6.

[12] Adam G, Rampášek L, Safikhani Z et al. Machine learning approaches to drug response prediction: challenges and recent progress. npj Precis Oncol. 2020;4:1–10. 10.1038/s41698-020-0122-1. 32566759 PMC7296033

[13] Ma J, Fong SH, Luo Y et al. Few-shot learning creates predictive models of drug response that translate from high-throughput screens to individual patients. Nat Cancer. 2021;2:233–44. 10.1038/s43018-020-00169-2. 34223192 PMC8248912

[14] Lotfollahi M, Klimovskaia Susmelj A, De Donno C et al. Predicting cellular responses to complex perturbations in high-throughput screens. Mol Syst Biol. 2023;19:e11517. 10.15252/msb.202211517. 37154091 PMC10258562

[15] Jiang W, Ye W, Tan X et al. Network-based multi-omics integrative analysis methods in drug discovery: a systematic review. BioData Min. 2025;18:27. 10.1186/s13040-025-00442-z. 40155979 PMC11954193

[16] Tran D, Nguyen H, Pham VD et al. A comprehensive review of cancer survival prediction using multi-omics integration and clinical variables. Brief Bioinform. 2025;26:150. 10.1093/bib/bbaf150. PMC11994034 40221959

[17] Karl F . A free energy principle for biological systems. Entropy. 2012;14:2100–21. 10.3390/e14112100. 23204829 PMC3510653

[18] Waltermann C, Klipp E. Information theory based approaches to cellular signaling. Biochim Biophys Acta. 2011;1810:924–32. 10.1016/j.bbagen.2011.07.009. 21798319

[19] Bowsher CG, Swain PS. Environmental sensing, information transfer, and cellular decision-making. Curr Opin Biotechnol. 2014;28:149–55. 10.1016/j.copbio.2014.04.010. 24846821

[20] Rockne RC, Branciamore S, Qi J et al. State-transition analysis of time-sequential gene expression identifies critical points that predict development of acute myeloid leukemia. Cancer Res. 2020;80:3157–69. 10.1158/0008-5472.CAN-20-0354. 32414754 PMC7416495

[21] Vasudevan S, Flashner-Abramson E, Remacle F et al. Personalized disease signatures through information-theoretic compaction of big cancer data. Proc Natl Acad Sci USA. 2018;115:7694–9. 10.1073/pnas.1804214115. 29976841 PMC6065026

[22] Szklarczyk D, Gable AL, Nastou KC et al. The STRING database in 2021: customizable protein–protein networks, and functional characterization of user-uploaded gene/measurement sets. Nucleic Acids Res. 2021;49:D605–12. 10.1093/nar/gkaa1074. 33237311 PMC7779004

[23] Pantziarka P, Capistrano IR, De Potter A et al. An Open Access Database of Licensed Cancer Drugs. Front Pharmacol. 2021;12:627574. 10.3389/fphar.2021.627574. 33776770 PMC7991999

[24] Ahmad S, Jose Da Costa Gonzales L, Bowler-Barnett EH et al. The UniProt website API: facilitating programmatic access to protein knowledge. Nucleic Acids Res. 2025;53:W547–53. 10.1093/nar/gkaf394. 40331428 PMC12230682

[25] Rao RD, Mladek AC, Lamont JD et al. Disruption of parallel and converging signaling pathways contributes to the synergistic antitumor effects of simultaneous mTOR and EGFR inhibition in GBM cells. Neoplasia. 2005;7:921–9. 10.1593/neo.05361. 16242075 PMC1502028

[26] Hoffman TE, Nangia V, Ill CR et al. Multiple cancers escape from multiple MAPK pathway inhibitors and use DNA replication stress signaling to tolerate aberrant cell cycles. Sci Signal. 2023;16:eade8744. 10.1126/scisignal.ade8744. 37527351 PMC10704347

[27] Vasudevan S, Flashner-Abramson E, Alkhatib H et al. Overcoming resistance to BRAF^V600E^ inhibition in melanoma by deciphering and targeting personalized protein network alterations. npj Precis Oncol. 2021;5:50. 10.1038/s41698-021-00190-3. 34112933 PMC8192524

[28] Flashner-Abramson E, Vasudevan S, Adejumobi IA et al. Decoding cancer heterogeneity: studying patient-specific signaling signatures towards personalized cancer therapy. Theranostics. 2019;9:5149–65. 10.7150/thno.31657. 31410207 PMC6691586

[29] Huang S, Wang H, Chen W et al. Tamoxifen inhibits cell proliferation by impaired glucose metabolism in gallbladder cancer. J Cell Mol Med. 2019;24:1599–613. 10.1111/jcmm.14851. 31782270 PMC6991689

[30] Nierengarten MB . FDA approves capivasertib with fulvestrant for breast cancer. Cancer. 2024;130:835–6. 10.1002/cncr.35238. 38396318

