# Supplementary material for: ProteinNetworkSight: a user-friendly platform for transforming co-expression patterns into actionable therapeutic insights through interactive network visualization
Source: Nucleic Acids Res. 2026 May 14;54(W1):W384–91. doi: 10.1093/nar/gkag477 (PMC13355079; doi:10.1093/nar/gkag477)
Supplement: gkag477_Supplemental_File [file gkag477_supplemental_file.docx]

**Supplementary Material for**

**ProteinNetworkSight: A User-Friendly Platform for Transforming Co-Expression Patterns into Actionable Therapeutic Insights Through Interactive Network Visualization**

Omri Nahor^1^, Nitzan Migdal^1^, Ayelet Gibli^#1^, Tohar Tsvitman^#1^, Aviv Eldad^1^, Shell Raveh^2^, Gil Polinovski^2^, Deema Zaid^2^, Nataly Kravchenko-Balasha^*2^ and Noa E. Cohen^*1^

^1^ School of Software and Electrical Engineering, Azrieli College of Engineering, Jerusalem, 9103501, Israel

^2^ The institute of Biomedical and Oral Research, The Hebrew University of Jerusalem, Jerusalem, [9103401](https://www.google.com/search?rlz=1C1GCEU_iwIL825IL825&q=9103401&stick=H4sIAAAAAAAAAONgVuLVT9c3NMyqMC82L04yecRowS3w8sc9YSn9SWtOXmPU5OIKzsgvd80rySypFJLmYoOyBKX4uVB18ixiZbc0NDA2MTAEABpW8KNWAAAA), Israel.

^#^ Equal contribution

^*^ Equal contributors and corresponding authors

# Supplementary File S1 – Demonstration of the Tool Using the Example File

An example of the web-based tool being used is displayed in Figure S1. The example input file available on the website, was derived from the supplementary data of the study which identified protein-protein co-expression patterns in 353 BRAFV600E and BRAFWT skin cutaneous melanoma (SKCM) samples and 372 thyroid carcinomas (THCA) (725 total samples) (1), which were initially profiled for 216 cancer-related proteins per sample (for the example file, 40 proteins were left).

The original dataset was characterized by 17 distinct protein-protein co-expression patterns that manifested in various patient groups. For simplicity, for creating the example file we fetch 5 columns only. A score (a weight that represents how each protein contributes to each pattern) was assigned to each protein. Proteins that fall into a specific range—in this example, > 0.1 and < -0.1 were considered to contribute to each network (1). ProteinNetworkSight provides a biological and clinical interpretation of all patterns at the same time, using 5 numerical vectors as input, which include protein names, scores for 40 proteins, and threshold limits.

The ProteinNetworkSight website generates a list of protein names recognized by STRING by first eliminating modifications (such as phospho) from protein names and comparing the resulting protein names to STRING names. The user is prompted with names that could not be found in STRING so the user can modify them. Subsequently, employing the threshold limits ProteinNetworkSight displays 5 networks at once, each of which reflects an input vector of scores. The names of the proteins have been restored back to display the protein names together with the matching modification (e.g., including phosphorylation) in each network. 13 red (negative scores) and 6 blue (positive scores) nodes, for instance, are part of the output network reflecting the *first* vector (named G1). The biggest radius belongs to TIGAR, which has the highest absolute value score. Additionally, the tabular representation shows the node degrees and final score calculations along with drugs associated with the protein. We can also see whether the original gene/protein name was used or an alternative name.

A scientist or clinician may choose a protein in each network with the greatest final score (a value calculated by combining the number of protein partners and the input score) and an FDA-approved drug for further research or therapy. In the example illustrated in Fig. S1, TIGAR has the highest input score (0.41), indicating a large contribution to the protein-protein co-expression pattern, but its final score (0.524) is significantly lower compared to others, due to the smaller number of protein-protein interactions (only 1). This information can be very useful for making treatment/experimental decisions. If a patient has *several* active networks within their tumor, several proteins will be chosen from those networks for combined treatment (1, 2).

**Figure S1**


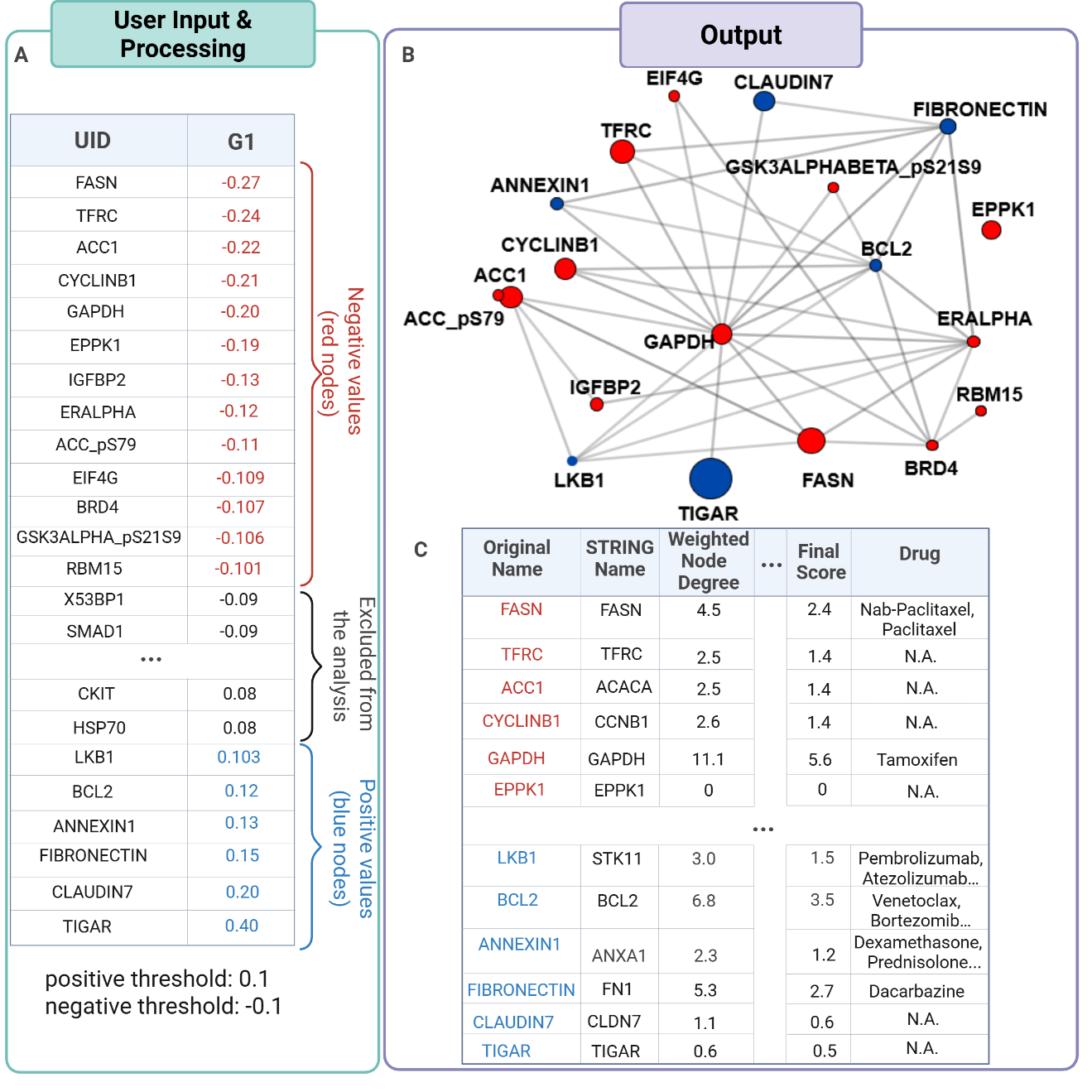


**Figure S1**. **Demonstration of the tool using real data**. **(A)** The example file (also available for download to help the user understand the needed format) has been uploaded. The file was extracted and adjusted (be deleting rows & columns) from [Supplementary Data 1](https://static-content.springer.com/esm/art%3A10.1038%2Fs41698-021-00190-3/MediaObjects/41698_2021_190_MOESM2_ESM.xlsx), G tab^1^. The input parameters inserted to the tool, along with the example input file are: “name of the 1st column indicating gene/protein name”: UID; “prefix of numerical columns indicating gene/protein scores”: G. The tool filters out genes/proteins whose score fall within the given range. Note that the user can define a different set of thresholds per score column. In this example, we utilized the following parameters: (1) “interaction STRING score threshold”: 0.4; (2) “positive threshold”: 0.1 (for positive scores) and “negative threshold”: -0.1 (for negative scores) since protein scores within this range (e.g. > 0.1 and < -0.1) had the largest impact on the network (1); (3) “organism”: homo sapiens. For demonstration purposes, scores are sorted and only the top 8 positive and bottom 15 negative proteins of the input are presented in (A) (out of a total of 40 proteins in the example input file).

In page 3 of the website (see **Figure S2**) the user is requested to adjust names of proteins not found in STRING, out of a list of suggestions. For ACC_pS79 and ACC1 we chose ACACA. Five gene names within this range (ANNEXIN1, ERALPHA, CLAUDIN7, CYCLINB1 and GSKALPHABETA, GSK3ALPHABETA_pS21S9) were not found in STRING and their names were set manually in page 4 of the input (we used ANXA1, ESR1, CLDN7, CCNB1, GSK3A and GSK3A respectively). The results for the first numerical column (G1 in this case) are displayed on the right panel as a network **(B)** or a table **(C)**, which includes information about the various scores generated by the tool (e.g., node degree, weighted node degree, and final score), as well as information about linked drugs. Created in BioRender. Cohen, N. (2026) <https://BioRender.com/h681fgc>.

**Figure S2**

| 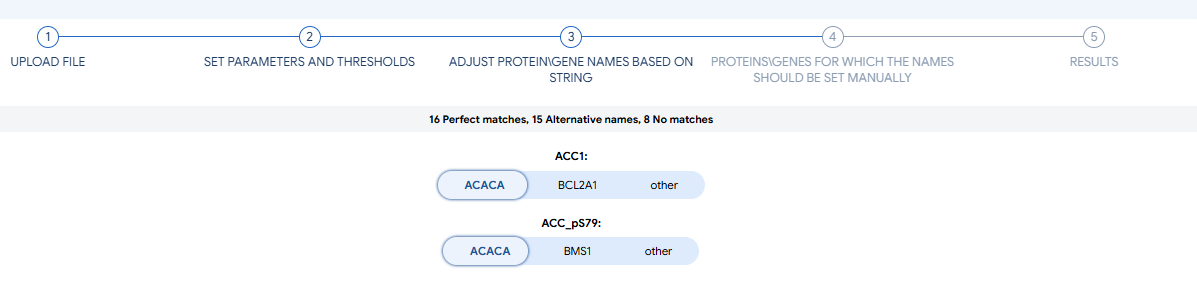  A |
| --- |
| B  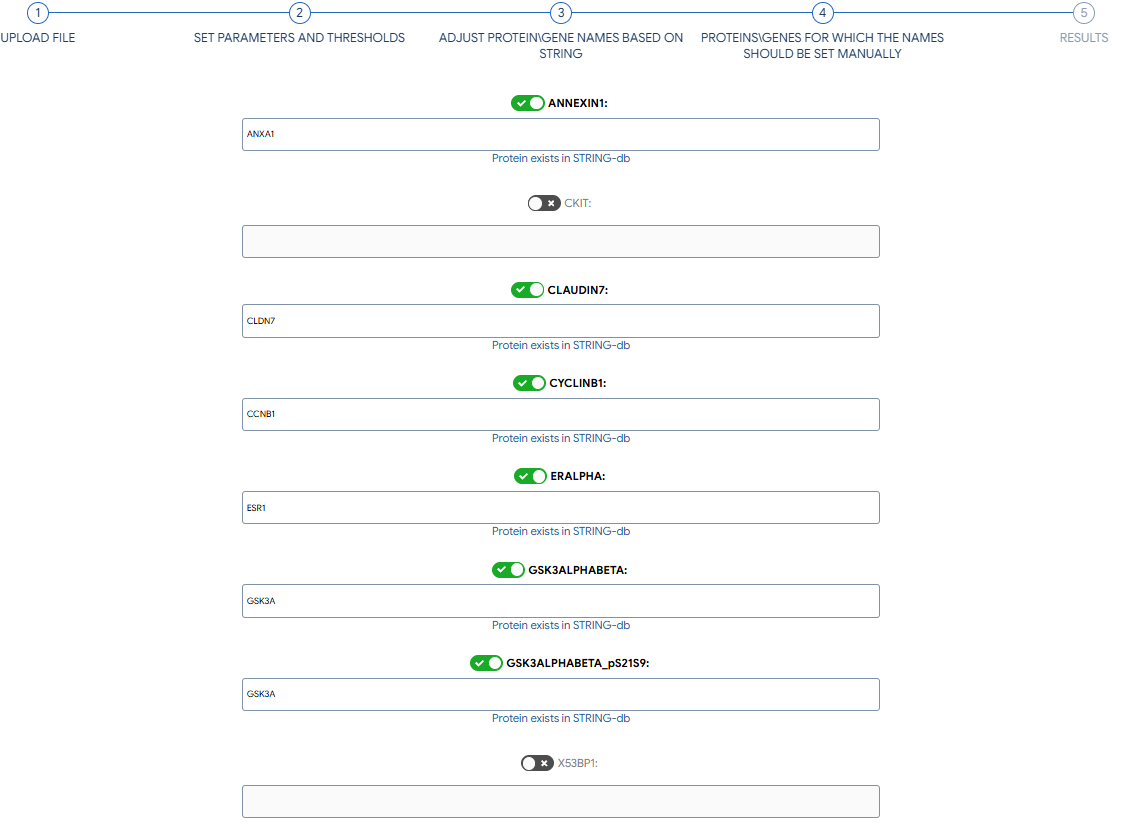 |

**Figure S2. A print screen of stages 3 (A) and 4 (B) from the website for the input example file.**

**Figure S3
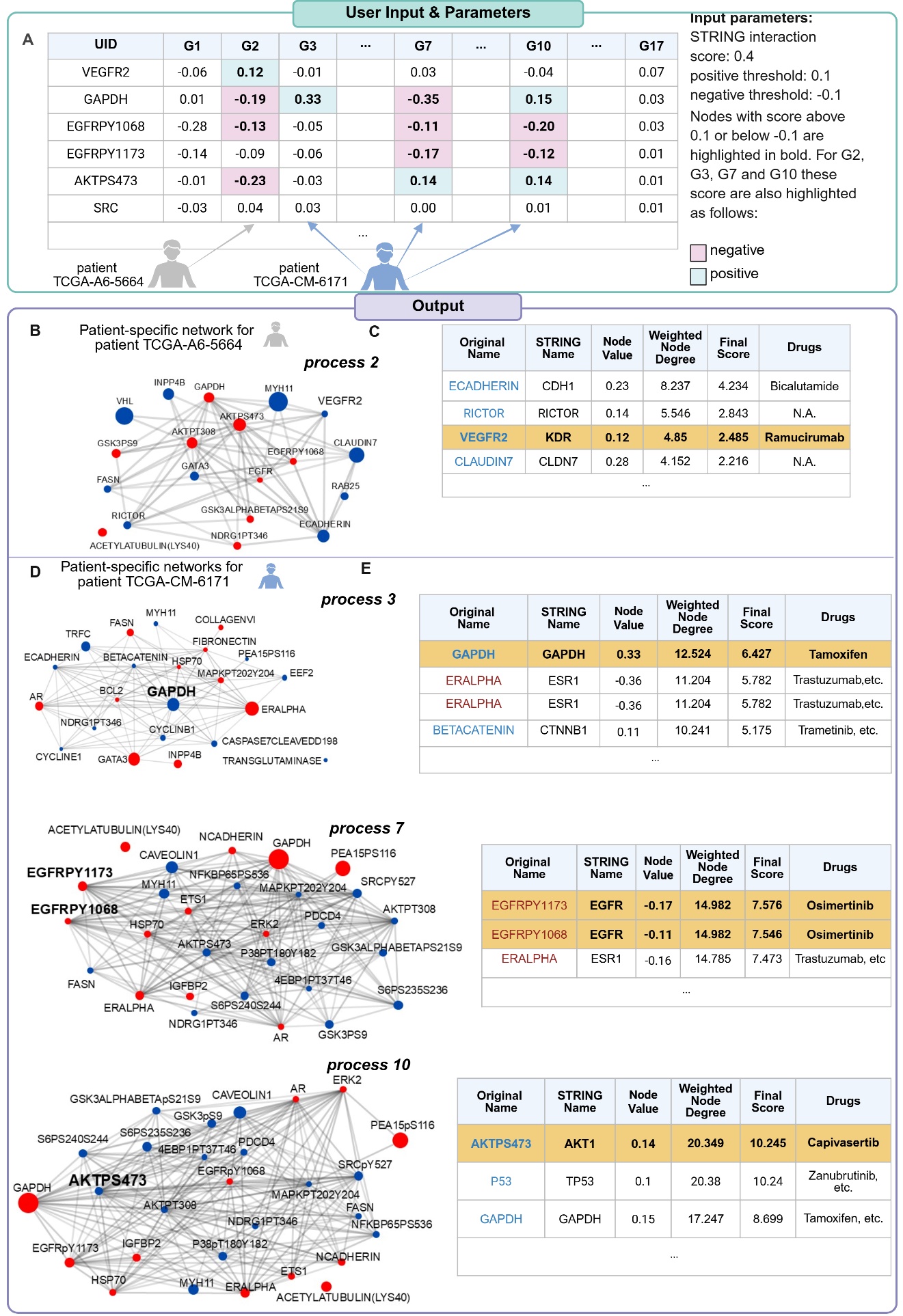
**

**Figure S3**. **Simultaneous multi-pattern processing resolving inter-tumor heterogeneity**. **(A)** The ProteinNetworkSight input interface demonstrating the simultaneous upload of 17 numeric vectors, representing the distinct biological patterns extracted from ~3,500 tumors across 11 cancer types (3). The column headers define the gene/protein identifiers ("UID") and the numerical score prefixes ("G"). The tool filters proteins based on user-defined thresholds; in this case, a STRING interaction score of 0.4 was applied. To focus on proteins with the highest network impact, thresholds were set at > 0.1 for positive scores and < -0.1 for negative scores, with the organism designated as Homo sapiens. For visualization purposes, only the 6 from the 181-protein dataset are displayed in the input panel for 6 score columns out of 17. We considered here two patients with the exact same disease (Colon Adenocarcinoma, COAD). Patient TCGA-CM-6171 is driven by processes 3, 7, and 10, while patient TCGA-A6-5664 is driven solely by process 2. **(B-E)** Calculated as detailed in (3), the G values reflect *coordinate deviations from the reference state*. *The signs and colors (red/blue) represent separation in signaling space*; importantly, a negative node value (e.g., in process 7) does not imply a reduction. The specific direction of change (increase/decrease) for these proteins was determined in the original computation (3), and those pre-calculated results serve as the basis for the patterns displayed here. *The central nodes (targets) highlighted in yellow within the output tables (****C, E****) specifically denote proteins that were computed to be upregulated in these individual patients, regardless of their G value sign (3).*

**(B–C)** Results for patient TCGA-A6-5664 (i.e., column G2) are presented as interactive networks (B) and data tables (C). These outputs detail calculated metrics - including node degree (not shown in the figure), weighted node degree, and final scores-alongside information regarding linked pharmacological agents; specifically, the output table for process 2 displays 4 out of 10 upregulated proteins co-expressed with VEGFR2. Here we present **(D–E)** Results for patient TCGA-CM-6171 (i.e., columns G3, G7 and G10) are presented as interactive networks (D) and data tables (E). The output tables for process 3 displays 2 out of 11 upregulated proteins co-expressed with GAPDH. The output tables for process 7 displays 3 out of 12 downregulated proteins co-expressed with EGFR (note that EGFRPY1173 and EGFRPY1068 are EGFR with two different modifications). The output tables for process 10 displays 3 out of 18 upregulated proteins co-expressed with AKTPS473 (AKT1). **(C, E)** The tabular output calculating the "Final Score" for the nodes within these specific patients' networks, highlighting how the platform maps completely different topological vulnerabilities to precise, off-label FDA-approved drugs (Tamoxifen, Osimertinib and Capivasertib for patient TCGA-CM-6171; Ramucirumab for patient TCGA-A6-5664).

In step 4, instead of the original name that was not identified in STRING, the matching STRING names were entered as follows: EIF4EBP1 for EIF4EBP1, ATAT1 for ACETYLATUBULIN(LYS40), AKT1 for AKTPS473, AKT1 for AKTPT308, BAK1 for BAK, CTNNB1 for BETACATENIN, CASP7 for CASPASE7CLEAVEDD198, CAV1 for CAVEOLIN1, CLDN7 for CLAUDIN7, COL6A1 for COLLAGENVI, CCNB1 for CYCLINB1, CCNE1 for CYCLINE1, CDH1 for ECADHERIN, EGFR for EGFRPY1068, EGFR for EGFRPY1173, EIF4G1 for EIF4G, ESR1 for ERALPHA, MAPK1 for ERK2, FN1 for FIBRONECTIN, GSK3A for GSK3ALPHABETAPS21S9, GSK3B for GSK3PS9, ERBB2 for HER2PY1248, HSPA4 for HSP70, MAPK3 for MAPKPT202Y204, CDH2 for NCADHERIN, NDRG1 for NDRG1PT346, RELA for NFKBP65PS536, MAPK14 for P38PT180Y182, TP53 for P53, RPS6KB1 for P70S6KPT389, PXN for PAXILLIN, PEA15 for PEA15PS116, RB1 for RBPS807S811, RPS6 for S6PS235S236, SRC for SRCPY527, TGM1 for TRANSGLUTAMINASE, TFRC for TRFC, KDR for VEGFR2, RPS6 for S6PS240S244. Created in BioRender. Cohen, N. (2026) <https://BioRender.com/wsk6jhy>.

**References**

1. Vasudevan,S., Flashner-Abramson,E., Alkhatib,H., Roy Chowdhury,S., Adejumobi,I.A., Vilenski,D., Stefansky,S., Rubinstein,A.M. and Kravchenko-Balasha,N. (2021) Overcoming resistance to BRAFV600E inhibition in melanoma by deciphering and targeting personalized protein network alterations. *npj Precis. Oncol.*, **5**, 50.
https://doi.org/10.1038/s41698-021-00190-3

2. Alkhatib,H., Conage-Pough,J., Roy Chowdhury,S., Shian,D., Zaid,D., Rubinstein,A.M., Sonnenblick,A., Peretz-Yablonsky,T., Granit,A., Carmon,E., *et al.* (2024) Patient-specific signaling signatures predict optimal therapeutic combinations for triple negative breast cancer. *Mol. Cancer*, **23**, 1–7.
https://doi.org/10.1186/s12943-023-01921-9
http://www.ncbi.nlm.nih.gov/pubmed/38229082

3. Flashner-Abramson,E., Vasudevan,S., Adejumobi,I.A., Sonnenblick,A. and Kravchenko-Balasha,N. (2019) Decoding cancer heterogeneity: Studying patient-specific signaling signatures towards personalized cancer therapy. *Theranostics*, **9**, 5149–5165.
https://doi.org/10.7150/thno.31657
